# Interventions to Enhance Adaptive Plasticity after Stroke: From Mechanisms to Therapeutic Perspectives

**DOI:** 10.1155/2016/9153501

**Published:** 2016-06-22

**Authors:** Adriana Conforto, Annette Sterr, Ela Plow, Leonardo Cohen

**Affiliations:** ^1^Neurology Clinical Division, Neurology Department, Hospital das Clinicas, Sao Paulo University, 05508-090 Sao Paulo, SP, Brazil; ^2^Hospital Israelita Albert Einstein, 05652-900 Sao Paulo, SP, Brazil; ^3^Department of Psychology, University of Surrey, Guildford, Surrey GU2 7XH, UK; ^4^Center for Neurological Restoration, Neurosurgery, Neurological Institute, Cleveland Clinic, Cleveland Clinic Foundation, Cleveland, OH 44195, USA; ^5^Human Cortical Physiology and Stroke Neurorehabilitation Section, National Institute of Neurological Disorders and Stroke, NIH, Bethesda, MD 20892, USA

Disability from stroke represents a great burden worldwide. The science of stroke rehabilitation flourished in the last decades, as the concept of brain plasticity advanced. It is now known that the adult brain can rewire in response to experience and training in healthy subjects and in disease states such as stroke. However, there is a gap between plasticity studies in animals demonstrating “rewiring” of neuronal circuits and the availability of neurorehabilitative strategies in the clinical domain. Although mechanisms underlying recovery are not completely understood, there is some evidence to suggest that recovery at least entails resolution of acute tissue damage and behavioral compensation, as well as functional and structural changes. There is evidence that better outcomes are associated with the greatest return of brain function toward the normal state of organization before stroke. How to drive adaptive reorganization remains an open question. In this special issue, five manuscripts address questions related to interventions that aim to enhance adaptive plasticity after stroke. How do we capitalize on mechanisms of plasticity to develop effective therapeutic approaches? How do we individualize therapy in such a heterogeneous condition as stroke?

Over the past decades, the interest in the potential use of brain stimulation to boost adaptive plasticity and enhance motor and language functions has steadily grown. According to one of the hypotheses of recovery after stroke, increased activity of the unaffected hemisphere and decreased activity of the affected hemisphere can be detrimental to motor and language performance. In this special issue, J. C. Griffis et al. assessed the effects of ten sessions of neuronavigated intermittent theta-burst stimulation (iTBS) given to residual left inferior frontal gyrus (IFG) in four patients with anomic aphasia and four patients with Broca's aphasia, in the chronic phase after left-hemisphere strokes. They intended to upregulate activity in the left IFG and decrease possibly detrimental hyperactivity in the right IFG. They went a step further and evaluated changes in connectivity between the right and left IFG after this intervention. Indeed, they observed a shift in the functional magnetic resonance imaging (fMRI) lateralization of IFG during a covert verb generation task after iTBS, as well as a decrease in connectivity between right and left IFG after treatment. Even though these preliminary results from a pilot, open-label study appear to be promising, larger, sham-controlled trials of iTBS are needed. A challenge in designing such trials is to define selection criteria that would help identify patients with the greatest likelihood of showing clinically meaningful improvements with brain stimulation treatment.

E. B. Plow et al. have suggested ways to address this challenge in the next manuscript. They have explained how one can personalize brain stimulation to improve motor outcomes for both patients with mild as well as patients with severe upper limb paresis. A model of individualized interventions that tailor treatments to each patient is proposed. The idea is to stratify paradigms of stimulation according to mechanisms underlying plasticity. Upregulation of excitability of the affected hemisphere and downregulation of excitability of the unaffected hemisphere according to the model of interhemispheric inhibition are not always beneficial. Therefore, strategies to match the right strategy to the right patient according to baseline level of severity of damage and paresis as well as structural or functional imaging were discussed. While emphasis of their discussion was on improving motor function, E. B. Plow et al. also discussed studies that extended the idea of tailoring interventions to treat language and mood disorders. The need to devise therapies that can decrease the burden of stroke in mildly affected patients, but especially in those with severe impairments, was highlighted. Still, models discussed in this manuscript have largely been theoretical; empirical investigations to test effectiveness of many models are still underway.

In a different manuscript, L. Furlan et al. reviewed studies that contributed to unveil the neural correlates of effects of upper limb immobilization and proposed that this intervention could be a valuable tool to develop novel strategies for patients with poor upper limb performance. Upper limb immobilization is one of the components of the original constraint-induced movement therapy (CMIT) protocol for stroke. CMIT has emerged as an evidence-based intervention to improve use of the paretic arm in relatively high-functioning subjects with stroke. This intervention has not yet been proven to benefit low-functioning patients, who present deep nonuse of the affected upper limb. However, upper limb immobilization can be employed as a model of low-functioning upper limb paresis and hence help to understand mechanisms underlying poor upper limp function, as well as refining treatments that can enhance outcomes in severely affected patients. Testing effects of motor imagery, action observation, peripheral somatosensory stimulation, and brain stimulation in the context of upper limb immobilization could advance the neurobehavioral framework and foster fine tunings in rehabilitation paradigms in order to potentiate adaptive plasticity as well as motor gains in these patients.

Similar to the notions suggested in manuscripts by J. C. Griffis et al. and E. B. Plow et al., where knowledge of mechanisms of plasticity is believed to be elemental to help match the right patient to the right treatment, M. Gandolla et al. investigated mechanisms of functional electrical stimulation (FES) treatment for foot drop in order to predict responsiveness to treatment. Some patients can relearn the ability to dorsiflex the ankle after finishing one month of gait rehabilitation aided by FES of the* tibialis anterior* muscle (“carryover”) while others cannot. The study aimed to evaluate mechanisms of the FES carryover effect by comparing differences in brain activity before FES treatment, in patients who presented a carryover effect, in patients who did not present this effect after treatment, and in controls without stroke, who did not undergo treatment. Brain areas of investigation were chosen based on previous knowledge about mechanisms underlying sensorimotor integration and voluntary movement. The authors were able to separate patients who evolved with and without a FES carryover effect after treatment, based on brain responses in the contralateral supplementary motor area (SMA) and angular gyrus. Furthermore, patients with a carryover effect, but not those without, had responses in SMA and in the primary motor cortex that were similar to those found in healthy controls. This manuscript is an example of how to translate data about mechanisms underlying plasticity into information that can be clinically useful, for instance, to help selecting patients in future trials in order to maximize potential treatment benefits. It also suggests that central plasticity can be induced by manipulating afferent input at the periphery, in line with other studies.

Another manuscript that evaluated effects of a peripheral intervention based on augmentation of somatosensory input in order to drive central motor plasticity was authored by C. Garcia et al. The authors applied electric currents to the skin overlying forearm muscles at four different frequencies in up to fourteen healthy subjects, for 5 minutes or 30 minutes. No changes in amplitudes of motor evoked potentials (MEPs), and no changes in H-reflexes were observed. The authors also studied five patients who had experienced upper limb spasticity in the chronic phase after stroke. Patients received 30 minutes of somatosensory stimulation delivered at 3 Hz. Again, the intervention failed to elicit changes in MEP amplitudes or spasticity tested during the performance of passive movement of the wrist. The results are very preliminary, considering the sample sizes, but suggest that short periods of electrical stimulation of the skin overlying forearm flexor muscles may not lead to measurable changes in cortical or spinal excitability or that if such changes occur, they are not captured by evaluation of MEPs or H-reflexes. Other peripheral interventions, such as somatosensory stimulation in the form of peripheral nerve stimulation or muscle vibration, seem more promising.

The articles published in this special issue underscore the importance of identifying the functional role of specific brain areas as well as brain networks that can assume functioning of lesioned tissue to develop interventions that can improve clinically significant outcomes. Effects of particular interventions not only on specific brain areas but also on interactions between areas should be tested in order to bridge the gap between animal studies, translational research, and clinical rehabilitation.

